# Diagnostic value of dipstick test in adult symptomatic urinary tract infections: results of a cross-sectional Tunisian study

**DOI:** 10.11604/pamj.2019.33.131.17190

**Published:** 2019-06-21

**Authors:** Foued Bellazreg, Maha Abid, Nadia Ben Lasfar, Zouhour Hattab, Wissem Hachfi, Amel Letaief

**Affiliations:** 1 Department of Infectious Diseases, Farhat Hached Hospital, Sousse, Tunisia

**Keywords:** Urinary tract infection, diagnosis, dipstick test, leukocyte esterase, nitrites

## Abstract

Urinary tract infections (UTIs) are common. The diagnosis is confirmed by urine culture which is costly and takes at least 24 hours before results are known. The aim of this study was to determine the diagnostic accuracy of dipstick test for the diagnosis of UTI in symptomatic adult patients. We conducted a cross-sectional study in the department of Infectious Diseases, Sousse-Tunisia during a two-year period. We included all patients with clinical signs of UTI. Urine samples were tested for the presence of leukocyte esterase (LE) and nitrites. Sensitivity, specificity, positive predictive value (PPV) and negative predictive value (NPV) of LE and nitrites were calculated against urine culture as gold standard. Four hundred thirty one patients, 139 men (32%) and 292 women (68%) were included. One hundred sixty six patients (39%) had UTI. The most frequently isolated microorganism was *Escherichia coli* (75%). LE had a high sensitivity (87%) but a low specificity (64%), while nitrites had a high specificity (95%) but a low sensitivity (48%). Combined positive LE and nitrites had a high PPV (85%) and combined negative LE and nitrites had a high NPV (92%), while positive LE combined with negative nitrites had a low PPV (47%) and a low NPV (53%). In conclusion, in adult patients with UTI symptoms, an alternate diagnosis should be considered if the LE is negative, while an early empirical antibiotic therapy against *Enterobacteriaceae* should be started if the nitrites are positive.

## Introduction

Urinary tract infections (UTIs) are common. They occur in 60% of women at least once in their lifetime and account for 23% of hospital-acquired infections [1, 2]. Clinical signs of UTIs lack of sensitivity and specificity and the definitive diagnosis is based on the isolation of a microorganism by urine culture. Since urine culture is costly and takes at least 24 hours before results are known, the use of simpler and faster diagnostic methods such as dipstick test should be useful to guide the initial diagnosis especially in an emergency context. In 2015, the Société de Pathologie Infectieuse de Langue Française recommended dipstick test as a diagnostic method in UTIs for its high negative predictive value (NPV) in women and its high positive predictive value (PPV) in men. Moreover, an English study has demonstrated that the use of urinary dipstick reduced the urine laboratory workload [3]. The aim of this study was to determine the diagnostic accuracy of dipstick test for the diagnosis of UTI in symptomatic adult patients [4].

## Methods

**Study design and population:** we conducted a cross-sectional study in the department of Infectious Diseases in Farhat Hached hospital, Sousse-Central Tunisia, during a two-year period. We included all hospitalized patients and outpatients, aged ≥ 18 years, suspected of having a UTI based on the presence of one or more among the following clinical signs: urinary frequency or urgency; dysuria, urinary retention lumbar; flank, iliac fossa, pelvic, or perineal pain; hematuria, mental confusion and fever. If another diagnosis such as acute appendicitis, salpingitis or endometritis was made, the patient was excluded from the study.

**Dipstick test and urine culture:** urine dipstick and urine culture were performed on fresh voided urine samples in all patients. Urine samples were tested for the presence of leukocyte esterase (LE) activity and nitrites using dipstick test according to the manufacturer's instructions, and expire dates were checked before use. Any change in color of the dipstick was considered a positive result and the values of positive results were (+), (++) or (+++) for LE and (+) for nitrites. Urine culture was processed in the Microbiology laboratory in our hopsital, within 2 hours of collection, according to the Comité de l'Antibiogramme de la Société Française de Microbiologie (CA-SFM) 2010 recommendations. It was considered positive if the specimen grew ≥ 10^5^ colony-forming units/ml of microorganisms. Specimens which cultures grew more than one microorganism were excluded from the study.

**Data analysis:** sensitivity, specificity, PPV and NPV of both LE and nitrites were calculated, separately and in combination, against urine culture as gold standard. All the parameters were compared between women and men. The following formulas were used: sensitivity = true positive / (true positive + false negative) x 100; specificity = true negative / (true negative + false positive) x 100; PPV = true positive / (true positive + false positive) x 100; NPV = true negative/(true negative + false negative) x 100. Data were entered and analyzed using the SPSS for Windows, version 10.0.

**Ethical considerations:** as our study didn't involve changes to the patients' usual medical management, no study protocol had been submitted to our hospital Ethics Committee approval. However, patients were provided with oral information and gave their verbal consent before being included in the study.

## Results

**Figure 1:**
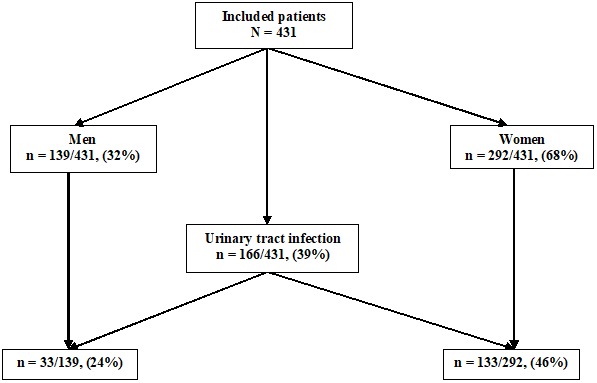
Distribution of urinary tract infections in our patients

Four hundred thirty one patients, 139 men (32%) and 292 women (68%) (sex-ratio = 0.48), mean age 48 years (17-84), were included in this study. One hundred sixty six patients (39%) had UTI, among them 33 (/139; 24%) were men and 133 (/292; 46%) were women. Thus, UTIs were more frequent in women than in men (p<0.001) (Figure 1). The isolated microorganisms were *Enterobacteriaceae* in 154 cases (93%), Gram positive cocci in 11 cases (6.5%) and *Pseudomonas aeruginosa* in one case (0.5%). The most frequently isolated microorganisms were *Escherichia coli (E. coli)* (75%) and *Klebsiella spp* (10%) (Table 1). Overall, the sensitivity of LE was 87% and the specificity was 64%, the sensitivity of nitrites was 48% and the specificity was 95%. The PPV and the NPV were 60% and 89% respectively for LE and 85% and 74% respectively for nitrites. LE was more specific in men (78%) compared to women (45%), and the NPV of nitrites was higher in men (85%) compared to women (68%) (Table 2). Overall, LE positivity combined with nitrites positivity had a PPV of 85%, LE negativity combined with nitrites negativity had a NPV of 92%, LE positivity combined with nitrites negativity had a PPV of 47% and LE negativity combined with nitrites positivity, noted in only 1.6% of patients, had a PPV of 86% (Table 3).

**Table 1:** Microorganisms isolated from urinary tract infections in our study

Microorganisms	Total N (%)	Women n (%)	Men n (%)
***Enterobacteriaceae***	154 (93%)*	124 (93)*	30 (91)*
*Escherichia coli*	124 (75%)	100 (75)	24 (73)
*Klebsiella spp*	17 (10)	14 (10.5)	3 (9)
Others	13 (7.5)	10 (7.5)	3 (9)
*Proteus mirabilis*	5 (3)	5 (4)	0
*Enterobacter spp*	4 (2.5)	2 (1.5)	2 (6)
*Citrobacter spp*	2 (1)	2 (1.5)	0
*Morganella spp*	2 (1)	1 (0.5)	1 (3)
**Gram positive cocci**	**11 (6.5)**	**9 (7)**	**2 (6)**
*Staphylococcus spp*	7 (4)	5 (4)	2 (6)
*Streptococcus spp*	3 (2)	3 (2)	0
*Enterococcus faecalis*	1 (0.5)	1 (1)	0
***Pseudomonas aeruginosa***	**1 (0.5)**	**0 (0)**	**1 (3)**
**Total**	**166**	**133**	**33**

* : percentages were calculated vertically ie. 154/166 (93%), 124/133 (93%) and 30/33 (91%)

**Table 2:** Diagnostic value of LE and nitrites in our study

Dipstick	Men			Women			Total		
	Urine culture			Urine culture			Urine culture		
	Positive n=33	Negative n=106	Total N=139	Positive n=133	Negative n=159	Total N=2922	Positive n=166	Negative n=265	Total N=4311
**LE**									
positive	26 (53)*	23 (47)*	49*	119 (58)*	87 (42)*	206*	145 (57)*	110 (43)*	255*
negative	7 (8)	83 (92)	90	14 (16)	72 (84)	86	21 (12)	155 (88)	176
**Nitrites**									
positive	15 (79)	4 (21)	19	64 (86)	10 (14)	74	79 (85)	14 (15)	93
negative	18 (15)	102 (85)	120	69 (32)	149 (68)	218	87 (26)	251 (74)	338
**LE**									
Sensitivity (%)	79			89			87		
Specificity (%)	78			45			64		
PPV (%)	53			58			57		
NPV (%)	92			84			88		
**Nitrites**									
Sensitivity (%)	45			48			48		
Specificity (%)	96			94			95		
PPV (%)	79			86			85		
NPV (%)	85			68			74		

LE: LeuKocyte esterase, PPV: positive predictive value, NPV: negative predictive value;

* : the percentages were calculated horizontally ie. 26/49 (53%), 119/206 (58%), 145/255 (57)

**Table 3:** Probability of urinary tract infection in terms of LE activity and nitrites

	Men			Women			Total		
	Urine culture			Urine culture			Urine culture		
Dipstick	Positive n (%)	Negative n (%)	Total N	Positive n (%)	Negative n (%)	Total N	Positive n (%)	Negative n (%)	Total N
**LE+ N+**	14 (78)	4 (22)	18	59 (87)	9 (13)	68	73 (85)	13 (15)	86
LE+ N+	4 (100)	0 (0)	4	8 (89)	1 (11)	9	12 (92)	1 (8)	13
LE++ N+	4 (50)	4 (50)	8	31 (86)	5 (14)	36	35 (80)	9 (20)	44
LE+++ N+	6 (100)	0 (0)	6	20 (87)	3 (13)	23	26 (90)	3 (10)	29
**LE+ N-**	12 (39)	19 (61)	31	60 (49)	63 (51)	123	72 (47)	82 (53)	154
LE+ N-	1 (11)	8 (89)	9	7 (32)	15 (68)	22	8 (26)	23 (74)	31
LE++ N-	8 (57)	6 (43)	14	39 (51)	38 (49)	77	47 (52)	44 (48)	91
LE+++ N-	3 (38)	5 (62)	8	14 (58)	10 (42)	24	17 (53)	15 (47)	32
**LE**- **N**-	6 (7)	83 (93)	89	9 (9)	86 (91)	95	15 (8)	169 (92)	184
**LE- N+**	1 (100)	0 (0)	1	5 (83)	1 (17)	6	6 (86)	1 (14)	7
**Total**	33	106	139	133	159	292	166	265	431

## Discussion

In the present study, LE had a high sensitivity (87%) and a high NPV (88%) but a low specificity especially in women (45%), while nitrites had a high specificity (95%) and a high PPV (85%) but a low sensitivity (48%). Combined positive LE and nitrites had a high PPV (85%) and combined negative LE and nitrites had a high NPV (92%), while positive LE combined with negative nitrites had a low PPV (47%) and a low NPV (53%). The diagnostic value of LE and of nitrites varied widely between the different studies. However, like in our study, a high NPV (87 to 93%) and a high specificity (94 to 99%) with low sensitivity (28 to 60%) were frequently noted for LE and nitrites respectively (Table 4). The differences between these studies may be explained by the heterogeneity of the patients' inclusion criteria (men or/and women; symptomatic or asymptomatic UTIs, cut-off used to define a positive urine culture) and by the important variation in the interpretation of dipstick test between practitioners [5-14]. For example, the rate of interobserver agreement in the interpretation of the LE test as proposed by the manufacturer between 37 general practitioners was only 68% [14]. Moreover, several factors can result in a false positive or a false negative result for LE or nitrites. Positive LE may occur in several conditions other than UTIs such as vulvovaginitis, urethritis, urinary lithiasis and diabetes mellitus, which may explain the low specificity of this test in UTIs [15]. The nitrite test detects the nitrate-reducing bacteria namely all the *Enterobacteriaceae* and most of the non-fermenters. Negative nitrite test in patients with positive urine culture may be noted when a UTI is caused by a microorganism that does not contain nitrite reductase or when the urine has been in the bladder for insufficiently long period (less than four hours). Lack of dietary nitrate and dilution of nitrite in urine may also explain false negative nitrite [11, 12, 16]. In our study, clinically suspected UTIs were confirmed by positive urine culture in only 38% of patients. They were more common in women (46%) than in men (24%), and the most frequently isolated microorganism was *E. coli.* In other studies, positive urine cultures in patients with clinically suspected UTIs varied considerably from 17 to 66%. This may be explained by the differences in inclusion criteria (women, men, or women and men; clinical signs) and in the cut off value (≥ 10^3^ colony forming units (CFU)/mL or ≥ 10^5^ CFU/mL) to diagnose a UTI in these studies [5-9]. Like in our study, *E. coli* was the most frequently (48 to 73.5%) isolated microorganism in UTIs in different studies [7-11].

**Table 4:** Diagnostic value of LE and nitrites in symptomatic urinary tract infections in different studies

Studies Dipstick	Zaman et al. Belgium (n = 420)	Gieteling et al. Netherlands (n = 104)	Sultana et al. Australia (n = 400)	Koeijers et al. Netherlands (n = 422)	Demilie et al. Ethiopia (n = 37)*	Our study Tunisia (n = 431)
Population study	men + women	men + women	men + women	men	women	men + women
**LE**						
Sensitivity (%)	74	69	72	82	71	87
Specificity (%)	76	92	86	53	90	64
PPV (%)	39	79	NA	NA	62	57
NPV (%)	93	87	NA	NA	93	89
**Nitrites**						
Sensitivity (%)	33	28	48	60	57	48
Specificity (%)	94	99	96	95	96	95
PPV (%)	52	79	NA	NA	80	85
NPV (%)	87	87	NA	NA	90	74

LE: leukocyte esterase; PPV: positive predictive value, NPV: negative predictive value; NA: not available;

* : the study included 367 patients, of whom 37 were symptomatic

## Conclusion

This study showed a high NPV of LE and a high PPV of nitrites in adult patients with UTI symptoms. Hence, in these patients, an alternate diagnosis should be considered if the LE is negative, while an early empirical antibiotic therapy against *Enterobacteriaceae* should be started if the nitrites are positive. In conclusion, when correctly performed, dipstick test should be a useful rapid diagnostic test for practitioners in their daily practice.

### What is known about this topic

- The rate of positive urine cultures in patients with clinically suspected urinary tract infections vary considerably between the studies;
- Leukocyte esterase has a high negative predictive value and a low specificity for the diagnosis of urinary tract infections;
- Nitrite test has a high positive predictive value and a low sensitivity for the diagnosis of urinary tract infections.

### What this study adds

- Urine cultures were positive in 39% of adult patients with clinical signs of urinary tract infections;
- Leukocyte esterase has a negative predictive value of 89% and a specificity of 64% in adult patients with clinical signs of urinary tract infections;
- Nitrite test has a positive predictive value of 85% and a sensitivity of 48% for the diagnosis of UTIs.

## Competing interests

The authors declare no competing interests.
